# Clinical impact of tumour burden on the efficacy of PD‐1/PD‐L1 inhibitors plus chemotherapy in non‐small‐cell lung cancer

**DOI:** 10.1002/cam4.5035

**Published:** 2022-07-18

**Authors:** Taichi Miyawaki, Hirotsugu Kenmotsu, Kosei Doshita, Hiroaki Kodama, Naoya Nishioka, Yuko Iida, Eriko Miyawaki, Nobuaki Mamesaya, Haruki Kobayashi, Shota Omori, Ryo Ko, Kazushige Wakuda, Akira Ono, Tateaki Naito, Haruyasu Murakami, Keita Mori, Hideyuki Harada, Masahiro Endo, Kazuhisa Takahashi, Toshiaki Takahashi

**Affiliations:** ^1^ Division of Thoracic Oncology Shizuoka Cancer Centre Shizuoka Japan; ^2^ Department of Respiratory Medicine Juntendo University Graduate School of Medicine Tokyo Japan; ^3^ Clinical Research Support Center Shizuoka Cancer Centre Shizuoka Japan; ^4^ Radiation and Proton Therapy Centre Shizuoka Cancer Centre Shizuoka Japan; ^5^ Division of Diagnostic Radiology Shizuoka Cancer Centre Shizuoka Japan

**Keywords:** clinical cancer research, immunology, lung cancer, metastasis, non‐small‐cell lung cancer

## Abstract

**Background:**

Programmed cell death 1 (PD‐1)/programmed cell death ligand (PD‐L1) inhibitors plus chemotherapy (ICI + Chemo) is the standard treatment for advanced non‐small‐cell lung cancer (NSCLC). However, the impact of tumour burden on the efficacy of ICI + Chemo remains unknown.

**Methods:**

We retrospectively evaluated 92 patients with advanced NSCLC treated with ICI + Chemo. Tumour burden was assessed as the sum of the longest diameter of the target lesion (BSLD) and number of metastatic lesions (BNMLs). We categorised the patients into three groups based on the combined BSLD and BNML values.

**Results:**

Sixty‐eight patients (74%) had progressive disease or died. Forty‐four patients (48%) in the low‐BSLD group had a median progression‐free survival (PFS) of 9.5 months, whereas patients in the high‐BSLD group had a median PFS of 4.6 months (hazard ratio [HR] = 0.54, *p* = 0012). Twenty‐five patients (27%) in the low‐BNML group had a median PFS of 9.6 months, whereas patients in the high‐BNML group had a median PFS of 6.5 months (HR = 0.51, *p* = 0.029). Low‐BSLD and low‐BNML were associated independently with improved PFS in multivariate analysis. Analysis of the tumour burden combined with BSLD and BNML revealed a trend towards improved PFS as the tumour burden decreased, with median PFS of 22.3, 8.7, and 3.9 months in the low‐ (*N* = 13), medium‐ (*N* = 42) and high‐burden (*N* = 37) groups respectively.

**Conclusions:**

Our findings demonstrated that a high tumour burden negatively impacts the efficacy of ICI + Chemo in patients with advanced NSCLC.

## Graphical abstract

Kaplan–Meier estimates of progression‐free survival (PFS) among patients with the low tumour burden, medium tumour burden and high tumour burden. The result of our study revealed a trend towards improved PFS as the tumour burden decreased.

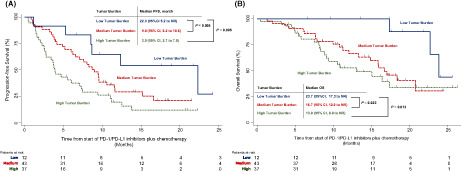

## 1 INTRODUCTION

Treatment with programmed cell death 1 (PD‐1) and programmed cell death ligand 1 (PD‐L1) inhibitors has become a key part of the therapeutic strategy for patients with advanced non‐small‐cell lung cancer (NSCLC).^1^, ^2^, ^3^, ^4^ Recent phase III clinical trials revealed that patients with advanced NSCLC being treated with PD‐1/PD‐L1 inhibitors plus chemotherapy had more favourable survival benefits than those receiving only chemotherapy as the first line of treatment.^5^, ^6^, ^7^

Despite their favourable survival outcomes, the median progression‐free survival (PFS) of patients with advanced NSCLC treated with PD‐1/PD‐L1 inhibitors plus chemotherapy is only 5–8 months.^5^, ^6^, ^7^ Thus, predictors of the response to PD‐1/PD‐L1 inhibitors plus chemotherapy must be identified to further improve the survival outcomes of patients. Basic research has demonstrated that an increased tumour burden is correlated with increased CD8 T cell exhaustion, which may reduce the therapeutic efficacy of PD‐1 inhibitors.^8^ Furthermore, previous clinical studies have showed that a high tumour burden is a negative predictive factor for the efficacy of PD‐1/PD‐L1 inhibitor monotherapy in patients with advanced NSCLC.^9^, ^10^ Nevertheless, it is unclear whether the tumour burden is also a negative predictive factor following treatment with PD‐1/PD‐L1 inhibitors plus chemotherapy. Therefore, we evaluated the clinical impact of tumour burden on the efficacy of PD‐1/PD‐L1 inhibitors plus chemotherapy in patients with advanced NSCLC.

Previous studies on solid cancers have focused on the baseline sum of the longest diameters (BSLD) of target lesions as a surrogate for tumour burden.^11^, ^12^, ^13^ However, a low number of metastases, defined as oligometastatic disease, has been associated with a low tumour burden and favourable prognosis.^14^, ^15^ Therefore, we evaluated both sum of the tumour diameter and number of metastases as surrogates for tumour burden to precisely investigate the impact of tumour burden on the efficacy of PD‐1/PD‐L1 inhibitor plus chemotherapy.

## 2 MATERIAL AND METHODS

### 2.1 Patients and data collection

We retrospectively collected data from the medical records of patients with advanced NSCLC who were treated with PD‐1/PD‐L1 inhibitors plus chemotherapy as the first‐line therapy at Shizuoka Cancer Center, Japan from December 2018 to March 2021. The following characteristics were analysed: age, sex, Eastern Cooperative Oncology Group performance status (ECOG‐PS), smoking history, histology, stage at diagnosis of lung cancer, PD‐L1 tumour proportion score (TPS) and metastatic sites. The study protocol was approved by the institutional ethical review board (registration number: J2020‐176‐2020‐1) and was performed in compliance with the principles of the Declaration of Helsinki. As this was a retrospective study, obtaining informed consent from the patients was not required. Immunohistochemical staining of PD‐L1 in tumour cells was performed using the pharmDx antibody (clone 22C3; Dako North America, Inc.).

### 2.2 Evaluating the tumour burden

All patients were assessed for all lesions in the chest, abdomen and intracranial region by computed tomography (CT) or magnetic resonance imaging (MRI) before the first administration of PD‐1/PD‐L1 inhibitors plus chemotherapy. In accordance with previous studies, BSLD was evaluated by measuring the target lesions based on the Response Evaluation Criteria for Solid Tumours (RECIST),^10^, ^12^, ^16^ and an optimal cut‐off threshold was defined as 76 mm to distinguish the high‐BSLD and low‐BSLD groups.^10^

The baseline number of metastatic lesions (BNML) was defined as any metastatic lesion identified on baseline CT or MRI scan, including both target and non‐target lesions. Metastatic thoracic lymph nodes (N1–N3) were collectively considered as a single metastatic lesion.^17^ Based on the criteria of oligometastatic disease, which represents a small number of metastases and low tumour burden, we defined patients with low‐BNML as those with 1–3 metastases and high‐BNML as those with four or more metastases.^14^, ^17^, ^18^

**FIGURE 1:**
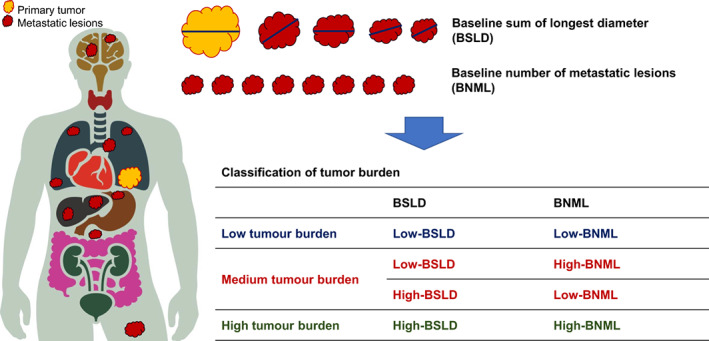
Measurement of tumour burden based on baseline sum of the target lesions' longest diameters and baseline number of metastatic lesions.

To estimate the impact of tumour burden more accurately, we classified the patients into three groups by combining the BSLD and BNML data. Patients with low‐BSLD and low‐BNML were categorised into the ‘low tumour burden’ group, those with low‐BNML/high‐BSLD or high‐BNML/low‐BSLD into the ‘medium tumour burden’ group, and those with high‐BSLD and high‐BNML into the ‘high tumour burden’ group (Figure 1).

PD was identified by reviewing the follow‐up radiological imaging data, including the data of CT and MRI, after the initiation of PD‐1/PD‐L1 inhibitors plus chemotherapy. Tumour responses were classified according to RECIST version 1.1.^19^

### 2.3 Statistical analysis

The correlation between BSLD and BNML was evaluated based on Spearman's rank correlation. PFS was defined as the time from the start of PD‐1/PD‐L1 inhibitor plus chemotherapy to disease progression or death. Overall survival (OS) was defined as the time from the start of PD‐1/PD‐L1 inhibitor plus chemotherapy to death. PFS and OS were estimated using the Kaplan–Meier method and compared using the log‐rank test. Potential risk factors were assessed using univariate and multivariate analyses with a Cox proportional hazards model for PFS. Covariates in the univariate analysis included BSLD, BNML, PD‐L1 TPS, age, sex, smoking status, ECOG‐PS, stage (Stage IV vs. recurrence), and initial central nervous system metastasis. Factors with *p* < 0.2 in the univariate analysis, initial stage, and PD‐L1 TPS, as known predictors of PD‐1/PD‐L1 inhibitors, were included in the subsequent multivariate analysis. All categorical variables were analysed using Fisher's exact test and continuous variables were analysed using the *t*‐test. All analyses were performed using STATA software (version 14.0; Stata Corp.).

## 3 RESULTS

### 3.1 Patient characteristics

Of the 120 consecutive patients with advanced NSCLC who were treated with PD‐1/PD‐L1 inhibitors plus chemotherapy, we excluded 28 patients who participated in clinical trials. Thus, we included 92 patients in the study. The clinical characteristics of the entire cohort are summarised in Table 1. Most patients were males (76%), showed ECOG‐PS of 1 (76%), and had a history of smoking (88%). Additionally, most tumours were non‐squamous cell carcinoma (90%) or initial stage IV (77%). Forty‐four patients (48%) had low‐BSLD and 48 (52%) had high‐BSLD. Twenty‐three (25%) patients had low‐BNML and 69 (75%) had high‐BNML. The proportion of patients with initial stage IV and PD‐L1 TPS ≥50% was higher in the high‐BSLD group than in the low‐BSLD group. In addition, the percent of patients with adrenal and distant lymph node metastases was higher in the high‐BSLD group than in the low‐BSLD group. Finally, the ratio of patients with pleural metastases was higher among patients in the low‐BSLD group than among those in the high‐BSLD group.

**TABLE 1:** Patient characteristics

Characteristics *N* = 92 (%)	Total *N* = 92 (%)	Low‐BSLD *N* = 44 (%)	High‐BSLD *N* = 48 (%)	*p*	Low‐BNML *N* = 23 (%)	High‐BNML *N* = 69 (%)	*p*
Median age (range)	67 (35–86)	67 (35–77)	66 (36–86)	0.653	67 (35–79)	67 (36–86)	0.842
Sex							
Male	70 (76)	32 (73)	38 (79)	0.469	15 (65)	55 (80)	0.158
Female	22 (24)	12 (27)	10 (21)		8 (35)	14 (20)	
ECOG‐PS							
0	22 (24)	14 (32)	8 (17)	0.089	3 (17)	19 (26)	0.158
1	70 (76)	30 (68)	40 (83)		20 (83)	50 (74)	
Smoking status							
Ever	81 (88)	37 (84)	44 (92)	0.263	19 (83)	62 (90)	0.354
Never	11 (12)	7 (16)	4 (8)		4 (17)	7 (10)	
Histology							
Non‐squamous	83 (90)	39 (89)	44 (92)	0.625	21 (92)	62 (90)	0.839
Squamous	9 (10)	5 (11)	4 (8)		2 (8)	7 (10)	
Stage							
IV	71 (77)	26 (59)	45 (94)	**<0.001**	17 (74)	54 (78)	0.667
Recurrence	21 (21)	18 (41)	3 (6)		6 (26)	15 (22)	
PD‐L1 TPS (%)							
≥50%	11 (13)	3 (7)	8 (17)	**0.035**	1 (4)	10 (14)	0.537
1%–49%	34 (41)	20 (45)	14 (29)		8 (35)	26 (38)	
≤1%	38 (46)	14 (32)	24 (50)		11 (48)	27 (39)	
Unknown	9 (10)	7 (16)	7 (14)		3 (13)	6 (9)	
Metastatic organ							
Pleura	30 (32)	19 (43)	11 (23)	**0.038**	0	30 (43)	
Pulmonary	24 (26)	10 (23)	14 (29)	0.482	4 (17)	23 (33)	0.273
Bone	32 (35)	15 (34)	17 (35)	0.894	7 (30)	25 (36)	0.613
Brain	26 (28)	11 (25)	15 (31)	0.506	6 (23)	20 (29)	0.789
Liver	10 (11)	3 (7)	7 (15)	0.232	2 (9)	8 (12)	0.699
Adrenal grand	13 (14)	2 (5)	11 (23)	**0.012**	3 (13)	10 (14)	0.863
Extra‐thoracic lymph node	16 (17)	4 (6)	12 (9)	**0.044**	4 (17)	12 (17)	1.000
Others	15 (16)	4 (9)	11 (23)	0.073	6 (26)	9 (13)	0.143
Number of metastatic lesions							
1–3	23 (25)	12 (27)	11 (23)	0.630			
≥4	69 (75)	32 (73)	37 (77)				
BSLD							
<75 mm	44 (48)				12 (52)	32 (46)	0.630
≥75 mm	48 (52)				11 (48)	37 (54)	
Treatment regimen							
CBDCA + PEM + Pembro	39 (42)	20 (46)	19 (40)		9 (39)	30 (43)	
CDDP + PEM + Pembro	38 (41)	18 (41)	20 (42)		8 (35)	30 (43)	
CBDCA + nab‐PTX + Atezo	5 (6)	1 (2)	4 (8)		4 (17)	1 (1)	
CBDCA + PTX + Atezo + BEV	1 (1)	0	1 (2)		0	1 (1)	
CBDCA + nab‐PTX + Pembro	8 (9)	5 (11)	3 (6)		2 (9)	6 (9)	
CBDCA + PTX + Pembro	1 (1)	0	1 (2)		0	1 (1)	

Abbreviations: Atezo, atezolizumab; BEV, bevacizumab; BNML, baseline number of metastatic lesions; BSLD, baseline sum of the target lesions' longest diameters; CBDCA, carboplatin; CDDP, cisplatin; ECOG, Eastern Cooperative Oncology Group; nab‐PTX, nab‐paclitaxel; PD‐L1, program death ligand 1; PEM, pemetrexed; Pembro, pembrolizumab; PS, performance statis; PTX, paclitaxel; TPS, tumour proportion score for PD‐L1 expression.

Significant *p*‐values shown in bold.

### 3.2 Analysis associated with BSLD and BNML

**FIGURE 2:**
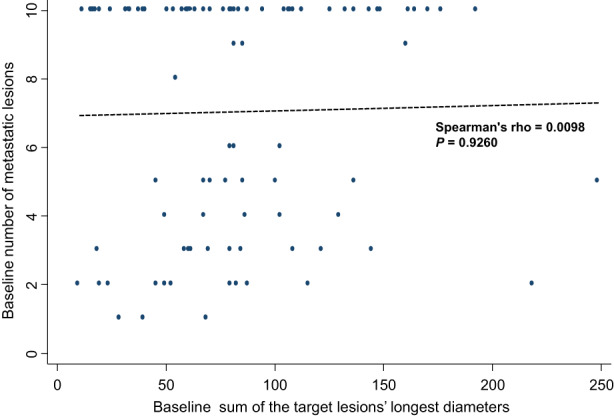
Correlation between the baseline sum of the target lesions' longest diameters and baseline number of metastatic lesions based on Spearman's rank correlation.

As shown in Figure 2, there was no significant correlation between BSLD and BNML (Spearman's rho = 0.0098, *p* = 0.926).

The objective response rate (ORR) was higher in patients with low‐BSLD than in those with high‐BSLD (57% and 40%, respectively; *p* = 0.098). The ORR improved significantly in patients with low‐BNML than those with high‐BNML (74% and 39% respectively; *p* = 0.004).

**FIGURE 3:**
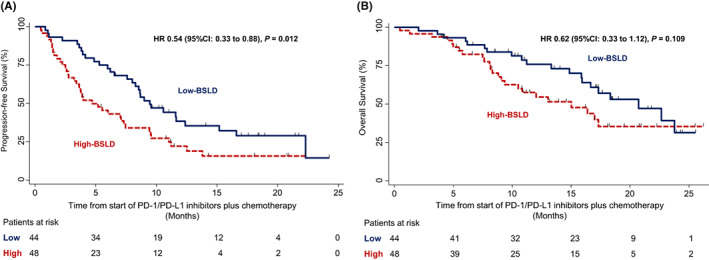
(A) Kaplan–Meier estimates of progression‐free survival among patients in the low‐ and high‐BSLD groups. HR, hazard ratio for disease progression or death. (B) Kaplan–Meier estimates of overall survival among patients in the low‐ and high‐BSLD groups. HR, hazard ratio for disease progression or death. BSLD, baseline sum of the target lesions' longest diameters; HR, hazard ratio for death.

The median follow‐up period for PFS was 15.6 months (range = 3.9–24.1 months). We observed 68 (74%) events of disease progression or death, and the median PFS was 7.6 months (95% confidence interval [CI] = 5.2–9.5) for all patients. The median PFS was 9.5 months (95% CI = 7.5–12.3) in patients with low‐BSLD and 4.6 months (95% CI = 3.2–7.3) in those with high‐BSLD, that is patients in the low‐BSLD group had a significantly better PFS (hazard ratio [HR] = 0.54; 95% CI = 0.33–0.88; *p* = 0.012; Figure 3A).

**FIGURE 4:**
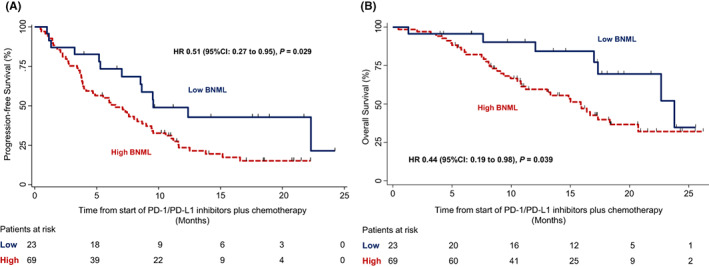
(A) Kaplan–Meier estimates of progression‐free survival among patients in the low‐ and high‐BNML groups. HR, hazard ratio for disease progression or death. (B) Kaplan–Meier estimates of overall survival among patients in the low‐ and high‐BNML groups. BNML, baseline number of metastatic lesions; HR, hazard ratio for death.

The median PFS was 9.6 months (95% CI = 7.0 to not reached [NR]) for patients in the low‐BNML group and 6.5 months (95% CI = 3.9–8.6) for those in the high‐BNML group, that is the patients in the low‐BNML group had a significantly improved PFS (HR = 0.51; 95% CI = 0.27–0.95; *p* = 0.029; Figure 4A).

The univariate analysis revealed that low‐BSLD (HR = 0.54; 95% CI = 0.33–0.88; *p* = 0.014) and low‐BNML (HR = 0.51; 95% CI = 0.27–0.94; *p* = 0.033) were favourable predictive factors for PFS. Additionally, a lack of central nervous system metastases tended to be a favourable predictive factor (HR = 0.66; 95% CI = 0.39–1.10; *p* = 0.108). In the multivariate analysis of PFS, low‐BSLD (HR = 0.55; 95% CI = 0.31–0.98; *p* = 0.042) and low‐BNML (HR = 0.48; 95% CI = 0.25–0.92; *p* = 0.026) were independent favourable predictive factors (Table 2).

**TABLE 2:** Predictor for PFS in PD‐1/PD‐L1 inhibitors plus chemotherapy

Covariates	Univariate analysis			Multivariate analysis		
	HR	95% CI	*p*‐value	HR	95% CI	*p*‐value
Low‐BSLD vs. high‐BSLD	**0.54**	**0.33–0.88**	**0.014**	**0.54**	**0.32–0.89**	**0.016**
Low‐BNML vs. High‐BNML	**0.51**	**0.27–0.94**	**0.033**	**0.49**	**0.25–0.92**	**0.026**
PD‐L1 TPS (≥50% vs. <50% or unknown)	0.83	0.36–1.94	0.679	0.53	0.22–1.25	0.147
Age (≥75 vs. < 75)	0.78	0.40–1.49	0.454			
Sex (female vs. male)	0.77	0.42–1.39	0.393			
Smoking (never vs. ever)	0.87	0.40–1.91	0.757			
Histology (non‐squamous vs. squamous)	0.64	0.30–1.35	0.246			
ECOG (PS 0 vs. 1)	0.79	0.45–1.38	0.398			
Stage (recurrence vs. stage IV)	0.71	0.39–1.27	0.244			
CNS metastases (present vs. absent)	0.66	0.39–1.10	0.108	0.71	0.41–1.21	0.204

Abbreviations: BNML, baseline number of metastatic lesions; BSLD, baseline sum of the target lesions' longest diameters; CI, confidence interval; ECOG, Eastern Cooperative Oncology Group; HR, hazard ratio; PD‐L1, program death ligand 1; PFS, progression‐free survival; PS, performance statis; TPS, tumour proportion score for PD‐L1 expression.

Significant *p*‐values shown in bold.

The median follow‐up period for OS was 16.5 months (range = 3.9–26.1 months). Forty‐four patients (48%) had died at the cut‐off date. The median OS was 17.3 months (95% CI = 14.8–23.7) for all patients. The median OS was 20.7 months (95% CI: 15.8–NR) for patients with low‐BSLD and 15.0 months (95% CI: 9.0–NR) for those with high‐BSLD. Although there was no significant difference, patients with low‐BSLD had a more favourable OS than those with high‐BSLD (HR = 0.62; 95% CI = 0.33–1.12; *p* = 0.109; Figure 3B).

The median OS was 23.7 months (95% CI: 16.9–NR) for patients with low‐BNML and 15.8 months (95% CI: 10.8–20.7) for those with high‐BNML, with patients in the low‐BNML group showing a significantly improved OS (HR = 0.44; 95% CI = 0.19–0.98; *p* = 0.039; Figure 4B).

### 3.3 Combined analysis of BNML and BSLD

To estimate the impact of tumour burden more accurately, we further classified the patients into low, medium, and high tumour burden groups based on the BSLD and BNML. Thirteen patients (14%) had a low tumour burden, 42 patients (46%) had a medium tumour burden and 37 patients (40%) had a high tumour burden. The ORR was significantly associated with the tumour burden according to this classification (85%, 50% and 32% for the three groups respectively; *p* = 0.005).

**FIGURE 5:**
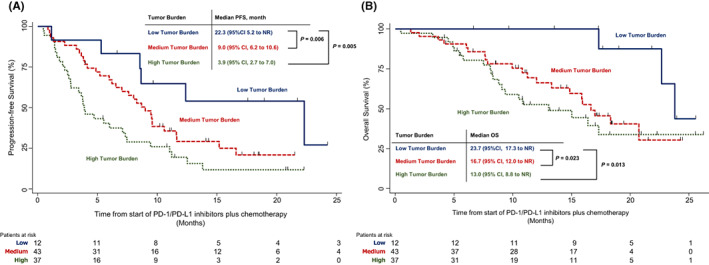
(A) Kaplan–Meier estimates of progression‐free survival among patients with the low tumour burden, medium tumour burden and high tumour burden. (B) Kaplan–Meier estimates of overall survival among patients with the low tumour burden, medium tumour burden and high tumour burden.

As shown in Figure 5A, the median PFS was 22.3 months (95% CI = 8.5 months to NR) in patients with a low tumour burden, 8.7 months (95% CI = 5.9–10.5 months) in patients with a medium tumour burden, and 3.9 months (95% CI = 2.7–7.0 months) in patients with a high tumour burden; a trend in PFS improvement with a decreasing tumour burden was observed. Patients with a low tumour burden had a significantly better PFS than those with a medium tumour burden (HR = 0.40; 95% CI = 0.15–1.03; *p* = 0.049) and those with high tumour burden (HR = 0.28; 95% CI = 0.10–0.72; *p* = 0.005).

As shown in Figure 5B, the median OS was 23.7 months (95% CI = 17.3 months to NR) in patients with a low tumour burden, 16.7 months (95% CI = 12.0 months to NR) in patients with a medium tumour burden, and 13.0 months (95% CI = 8.8 months to NR) in patients with a high tumour burden. Patients with a low tumour burden had a significantly better OS than those with a medium tumour burden (HR = 0.51; 95% CI = 0.27–0.86; *p* = 0.019) and high tumour burden (HR = 0.24; 95% CI = 0.68–0.81; *p* = 0.013).

## 4 DISCUSSION

To the best of our knowledge, this is the first study to show the impact of tumour burden on the efficacy of PD‐1/PD‐L1 inhibitors plus chemotherapy in patients with advanced NSCLC. The results of our study revealed that a high tumour burden had a negative impact on the efficacy of PD‐1/PD‐L1 inhibitors plus chemotherapy, regardless of other clinical confounding factors. Our results also showed that there was no correlation between BSLD and BNML, and that these two indices complemented each other as indicators of the tumour burden. Furthermore, combined analysis of BNML and BSLD suggested that PD‐1/PD‐L1 inhibitors plus chemotherapy is the most effective when the tumour burden is the lowest. Therefore, simultaneous assessment of BSLD and BNML may reveal the actual tumour burden.

As previously established, PD‐1/PD‐L1 inhibitors plus chemotherapy is not effective for all patients with advanced NSCLC; even in patients in whom this treatment is effective, a durable response is not necessarily achieved.^6^, ^7^, ^20^, ^21^ Therefore, to further improve the OS of patients, it is essential to identify patient populations that do not respond to PD‐1/PD‐L1 inhibitors plus chemotherapy or those showing difficulties in achieving a durable response to this therapeutic modality. Apart from PD‐L1 expression in tumours, a few other biomarkers have been identified for PD‐1/PD‐L1 inhibitors plus chemotherapy.^22^ Our study demonstrated that the tumour burden can be used as a clinical biomarker for the efficacy of PD‐1/PD‐L1 inhibitors plus chemotherapy, thereby providing valuable clinical implications for patients being administered this therapy.

A previous study on cytotoxic chemotherapy for advanced NSCLC has showed that BSLD is significantly associated with OS but not with PFS or the response rate.^12^ In the present study, we demonstrated that the tumour burden was significantly associated with the OS, PFS, and response rate following PD‐1/PD‐L1 inhibitors plus chemotherapy. Therefore, the tumour burden may be useful not only as a prognostic factor, but also as a predictor of the response of PD‐1/PD‐L1 inhibitors plus the chemotherapy response.

Additionally, several studies have indicated that elevated CD8^+^ tumour‐infiltrating lymphocytes (TILs) are an essential component of the response to PD‐1/PD‐L1 inhibitor monotherapy.^23^, ^24^ Thus, a high tumour burden may obstruct infiltration of the TILs.^25^, ^26^ Previous studies of several types of solid cancers have also revealed that tumour burden is associated with the efficacy of PD‐1/PD‐L1 inhibitor monotherapy, supporting our hypotheses.^9^, ^10^, ^11^, ^27^ Our results confirmed that the tumour burden can be used to determine the efficacy of treatment with PD‐1/PD‐L1 inhibitors plus chemotherapy in patients with advanced NSCLC.

A basic study revealed that tumour debulking improves the outcomes of PD‐1 inhibitor treatment.^28^ Thus, debulking tumour burden may maximise synergistic effects with PD‐1 inhibitors in patients with NSCLC. As the clinical features of tumour burden also apply to PD‐1/PD‐L1 inhibitors plus chemotherapy, it is possible that reducing the tumour burden can enhance the therapeutic effect of PD‐1/PD‐L1 inhibitors plus chemotherapy in patients with advanced NSCLC.

There were some limitations to our study. First, our analysis was limited because of the retrospective nature of our study and our inability to account for unknown confounders. The small number of patients in this study may have affected the statistical power. Furthermore, the study was conducted in a single cohort from a single institution and was not independently validated. Unlike the results of previous studies, high expression of PD‐L1 was not an independent predictive factor for the efficacy of PD‐1/PD‐L1 inhibitors plus chemotherapy in this study. Although patients with high PD‐L1 expression have been mainly treated with pembrolizumab monotherapy in our institution, those with a high tumour burden were treated with PD‐1/PD‐L1 inhibitors plus chemotherapy. This bias in treatment selection may have led to PD‐L1 expression in tumours not being a predictor of efficacy of PD‐1/PD‐L1 inhibitors plus chemotherapy in the present study. Furthermore, we could not directly evaluate tumour burden, as there is no common threshold for BNML or BSLD to estimate the tumour burden. However, this study and previous studies demonstrated the clinical relevance of the tumour burden in chemotherapy, PD‐1/PD‐L1 inhibitor use, and PD‐1/PD‐L1 inhibitor plus chemotherapy using a common threshold for BSLD.^10^, ^12^ For BNML, we used the criteria for oligometastatic disease,^14^, ^17^, ^18^ which has important clinical significance and is a clinically meaningful threshold. Despite the limitations outlined above, we believe that the results of this study are consistent with those of previous studies and are scientifically reasonable.

In conclusion, our findings suggest that a high tumour burden has a negative impact on the efficacy of PD‐1/PD‐L1 inhibitor plus chemotherapy in patients with advanced NSCLC. The addition of local ablative therapy for primary and metastatic sites to PD‐1/PD‐L1 inhibitors plus chemotherapy may improve survival outcomes by reducing the tumour burden, which should be further evaluated in prospective trials.

## AUTHOR CONTRIBUTIONS

Taichi Miyawaki: Conceptualisation, data curation, investigation, methodology, visualisation, writing—original draft. Hirotsugu Kenmotsu: Conceptualisation, Investigation, Methodology, Project administration, Supervision, Writing—review & editing. Kosei Doshita: Investigation. Hiroaki Kodama: Investigation. Naoya Nishioka: Investigation. Yuko Iida: Investigation. Eriko Miyawaki: Investigation. Nobuaki Mamesaya: Investigation. Haruki Kobayashi: Investigation. Shota Omori: Investigation. Ryo Ko: Investigation. Kazushige Wakuda: Investigation. Akira Ono: Investigation. Tateaki Naito: Conceptualisation, Investigation. Haruyasu Murakami: Investigation. Keita Mori: Methodology, Formal analysis, Supervision. Hideyuki Harada: Supervision. Masahiro Endo: Supervision. Kazuhisa Takahashi: Supervision. Toshiaki Takahashi: Project administration, Supervision, Writing—review & editing.

## FUNDING INFORMATION

This research did not receive any specific grant from funding agencies in the public, commercial, or not‐for‐profit sectors.

## CONFLICT OF INTEREST

Dr. Kenmotsu reports grants and personal fees from Chugai Pharmaceutical Co., Ltd., personal fees from Ono Pharmaceutical Co., Ltd., personal fees from Boehringer Ingelheim, personal fees from Eli Lilly K.K, personal fees from Kyowa Hakko Kirin Co., Ltd., personal fees from Bristol‐Myers Squibb, personal fees from MSD, grants and personal fees from Novartis Pharma K.K., grants and personal fees from Daiichi‐Sankyo Co., Ltd., grants and personal fees from AstraZeneca K.K., personal fees from Pfizer, personal fees from Taiho Pharma, outside the submitted work.

Dr. Mamesaya reports personal fees from AstraZeneca K.K., Pfizer Japan, Inc., personal fees from Chugai Pharmaceutical Co., Ltd., grants and personal fees from Boehringer Ingelheim, personal fees from MSD K.K., personal fees from Taiho Pharmaceutical Co., Ltd., personal fees from Oon Pharmaceutical Co., Ltd., outside the submitted work.

Dr. Kobayashi reports personal fees from Eli Lilly K.K, personal fees from Taiho Pharmaceutical, personal fees from AstraZeneca, outside the submitted work.

Dr. Omori reports personal fees from Chugai Pharmaceutical Co., Ltd., Ono Pharmaceutical, AstraZeneca K.K., Boehringer Ingelheim, Taiho Pharmaceutical, and MSD, which are unrelated to the submitted work.

Dr. Ko reports grants and personal fees from Boehringer Ingelheim, grants and personal fees from AstraZeneca, personal fees from Taiho Pharmaceutical, personal fees from Chugai Pharmaceutical, personal fees from Ono Pharmaceutical, personal fees from Pfizer, personal fees from Lilly, outside the submitted work.

Dr. Wakuda reports grants and personal fees from Chugai Pharmaceutical Co., Ltd., personal fees from Taiho Pharmaceutical, personal fees from Boehringer Ingelheim, personal fees from Eli Lilly K.K., personal fees from Ono Pharmaceutical, personal fees from MSD, grants and personal fees from AstraZeneca, grants from Novartis, grants from Abbvie, outside the submitted work.

Dr. Ono reports grants from Taiho Pharmaceutical, grants from Ono Pharmaceutical, grants from Chugai Pharmaceutical Co., Ltd., grants from Novartis Pharma K.K., outside the submitted work.

Dr. Murakami reports personal fees from AstraZeneca K.K., Ono Pharmaceutical, Bristol‐Myers Squibb Japan, Chugai Pharmaceutical Co., Ltd., Pfizer Inc., Novartis Pharma K.K., Boehringer Ingelheim, Taiho Pharmaceutical, Eli Lilly K.K., and MSD, which are unrelated to the submitted work.

Dr. Harada reports personal fees from Daiichi‐Sankyo Pharmaceutical Co. during the conduct of the study as well as personal fees from Daiichi‐Sankyo Pharmaceutical Co., AstraZeneca K.K., Brain Labo Co., and Chugai Pharmaceutical Co. and grants from the Japan Agency for Medical Research and Development and the National Cancer Center Research and Development Fund, which are unrelated to the submitted work.

Dr. Endo reports personal fees from Ono Pharmaceutical, personal fees from AstraZeneca, personal fees from Takeda Pharmaceutical Co., Ltd., personal fees from Daiichi‐Sankyo Co., Ltd., outside the submitted work.

Dr. Kazuhisa Takahashi reports grants and personal fees from AstraZeneca K.K., Pfizer Japan, Inc., Eli Lilly K.K., MSD, and Boehringer Ingelheim as well as grants from Takeda Pharmaceutical Co., Ltd., Chugai Pharmaceutical Co., Ltd., Taiho Pharmaceutical Co., Ltd., KYORIN Pharmaceutical Co., Ltd., Ono Pharmaceutical Co., Ltd., GlaxoSmithKline Consumer Healthcare Japan K.K., Shionogi & Co., Ltd., and Novartis Pharma K.K., which are not related to the submitted work.

Dr. Toshiaki Takahashi reports grants and personal fees from AstraZeneca K.K., Pfizer Japan, Inc., grants and personal fees from Eli Lilly Japan K.K., grants and personal fees from Chugai Pharmaceutical Co., Ltd., grants and personal fees from Ono Pharmaceutical Co., Ltd., grants and personal fees from MSD K.K., grants and personal fees from Boehringer Ingelheim Japan, Inc., grants and personal fees from Pfizer Japan, Inc., personal fees from Roche Diagnostics K.K., outside the submitted work.

The remaining authors declare no conflict of interest.

## ETHICS APPROVAL STATEMENT

The study protocol was approved by the institutional ethical review board (registration number: J2020‐176‐2020‐1) and was performed in compliance with the principles of the Declaration of Helsinki.

## PATIENT CONSENT STATEMENT

As this was a retrospective study, obtaining informed consent from the patients was not required.

## Data Availability

All data and material are available on reasonable request.
